# The association of postoperative global femoral offset with total hip arthroplasty outcomes

**DOI:** 10.1038/s41598-023-28863-y

**Published:** 2023-01-28

**Authors:** Yuki Hirano, Norio Imai, Asami Nozaki, Yoji Horigome, Hayato Suzuki, Hiroyuki Kawashima

**Affiliations:** 1 Division of Orthopedic Surgery Department of Regenerative and Transplant Medicine, Niigata University Graduate School of Medical and Dental Sciences, 1-757, Asahimachi-do-ri, Chuou ku, Niigata, Niigata 951-8510 Japan; 2 Division of Comprehensive Musculoskeletal Medicine, Niigata University Graduate School of Medical and Dental Sciences, 1-757, Asahimachi-do-ri, Chuou ku, Niigata, Niigata 951-8510 Japan

**Keywords:** Medical research, Pathogenesis

## Abstract

Global femoral offset (GFO) and femoral offset (FO) reportedly affect outcomes following total hip arthroplasty (THA). However, FO assessed using plain radiography is affected by internal and external rotations of the hip joint. We investigated the relationship between leg length discrepancy and Harris hip score (HHS), and their influence on acetabular offset (AO), FO, GFO, anterior femoral offset, and outcomes after THA. We retrospectively evaluated 140 patients with hip osteoarthritis who underwent THA. A three-dimensional (3D) pelvis and femur model created from computed tomography (data using ZedHip software was used to investigate these parameters. The modified (m)HHS scores were significantly improved from 49.0 to 88.8 in total mHHS, 20.0–44.5 in pain, and 28.9–44.4 points in function. Significant correlations were found between the differences in AO, FO, GFO, and pain score in binominal, with maximum values of − 1.24, + 1.54, and + 0.90 mm/100 cm body height, respectively. The maximum value of GFO and mHHS in binominal was + 1.17 mm/100 cm body height (BH). The optimal range of difference of GFO was − 1.75 to 4.09 mm/100 cm BH. This is the first report using a 3D method for assessing FO. Preoperative planning using the system could improve postoperative function.

## Introduction

Total hip arthroplasty (THA) is a widely performed procedure for hip arthritis that reliably relieves pain and improves function. However, there are cases of residual pain and insufficient functional improvement, and efforts continue to improve the postoperative outcomes. Global femoral offset (GFO) and femoral offset (FO) affect outcomes following total hip arthroplasty (THA), such as the hip function and activities of daily living. Mahmood et al. reported significant abduction muscle weakness and a tendency to use walking aids if the GFO decreased by more than 5 mm post THA^1^. Cassidy et al. reported a significantly low score in the postoperative WOMAC physical function test in a decreased femoral offset group^2^. Conversely, Esbjörnsson et al. reported that pain and quality of life were improved, and Bonnin et al. reported that the stress of the abductor muscles decreased when the acetabular component was placed slightly medially and the FO was enlarged^3,4^. Most of these reported outcomes are based on plain radiographs, which do not show the effects of hip internal and external rotation on FO^5^. In general, the FO is larger in internal rotation and smaller in external rotation; therefore, assessment by plain radiographs is not reproducible. The same can be said for the use of plain radiographs in preoperative planning. This is because hip rotation on preoperative plain radiographs is not necessarily reflected on postoperative plain radiographs. In addition, it is not possible to assess the anterior femoral offset (AFO) in the anteroposterior direction in an axial section^6^. Therefore, even though it is possible to predict the outcome after THA, it is difficult to use assessments obtained by these methods to plan preoperative component placement, including stem anteversion. In addition, AFO in the anteroposterior direction cannot be assessed using plain radiographs; hence, assessment using a unified coordinate system is necessary. Li et al. reported a lower walking speed and reduced stride length during gait in patients with a larger leg length discrepancy (LLD) after THA^7^; therefore, LLD may affect the postoperative outcomes. The purpose of this study was to investigate the relationship between LLD and Harris Hip Score (HHS), and their influence on acetabular offset (AO), FO, GFO, AFO, and outcome after THA. The optimal offsets and ranges to improve postoperative outcomes following total hip arthroplasty were determined based on measurements.

## Methods

### Patients

We enrolled patients who underwent THA between January 1, 2010, and December 31, 2019, in our institution. During the survey period, 376 THAs were performed, and we enrolled patients with hip osteoarthritis (HOA), in whom the center–edge angle of the non-surgical side of the hip joint was more than 25°, and asymptomatic. Patients with a history of lumbar, knee, and lower leg surgeries, severe knee OA, and severe acetabular dysplasia, such as Crowe type 3 or 4, were excluded. Finally, 140 patients (sex, 108 women and 32 men) were included in the study (Table 1). All THAs were performed using an anterolateral supine approach^8^ by seven experienced orthopedic surgeons. We attempted placement of the acetabular component to restore the original hip joint center position 40° at radiographic inclination and 15° at radiographic anteversion^9^ relative to the functional pelvic plane (FPP). This preserves the individual sagittal inclination of the anterior pelvic plane (APP), the plane including the most anterior point of the pubic symphysis and bilateral anterior superior iliac spine in the supine position. The stem was placed at 20°–25° anteversion relative to the retrocondylar plane (the plane including the most posterior points of the greater trochanter and bilateral femoral condyles)^10^ and was adjusted to the shape of the medullary canal of the femoral shaft using the combined anteversion theory^11,12^. This study was approved by the Institutional Review Board of Niigata University Graduate School of Medical and Dental Sciences (2019-0051). Informed consent for the study was waived by the Ethical Review Board of Niigata University School of Medicine because it was a cross-sectional, retrospective study without any intervention. All methods were performed in accordance with the relevant guidelines and regulations.

**Table 1:** Details of the participants.

Sex (male/female)	32/108
Age (years)*	56.2 ± 10.2 (34–77)
Surgical side (right/left)	83/57
Primary disease	DDH: 116 Idiopathic osteonecrosis of the femoral head: 22 Primary HOA: 2

*DDH* developmental dysplasia of the hip, *HOA* hemilateral hip osteoarthritis.

*Mean ± standard deviation (range).

### Measurement

We used ZedHip software (Lexi, Tokyo, Japan) to create a three-dimensional (3D) digital bone model from computed tomography (CT) data taken for preoperative 3D-planning and assessment of implant placement at 1 week after surgery^13,14^. A multi-slice CT scanner with a 64-row detector (Aquilion 64TM, Toshiba Medical Systems, Otawara, Tochigi, Japan) was used to acquire approximately 600 slices (slice thickness, 1.25 mm) from each limb.

**Figure 1:**
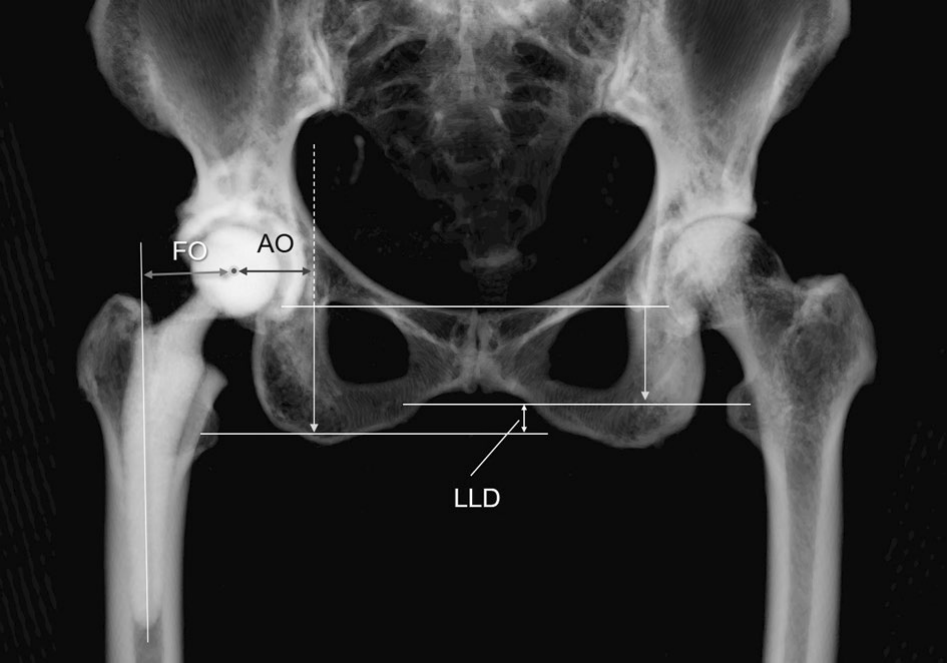
The measurement of AO, FO, and LLD. AO is the distance between the center of the femoral head and the medial wall of the teardrop. FO is the distance between the center of the femoral head and the anatomical axis of the femur. LLD is the difference between the surgical and nonsurgical sides. *AO* acetabular offset, *FO* femoral offset, *LLD* leg length discrepancy.

**Figure 2:**
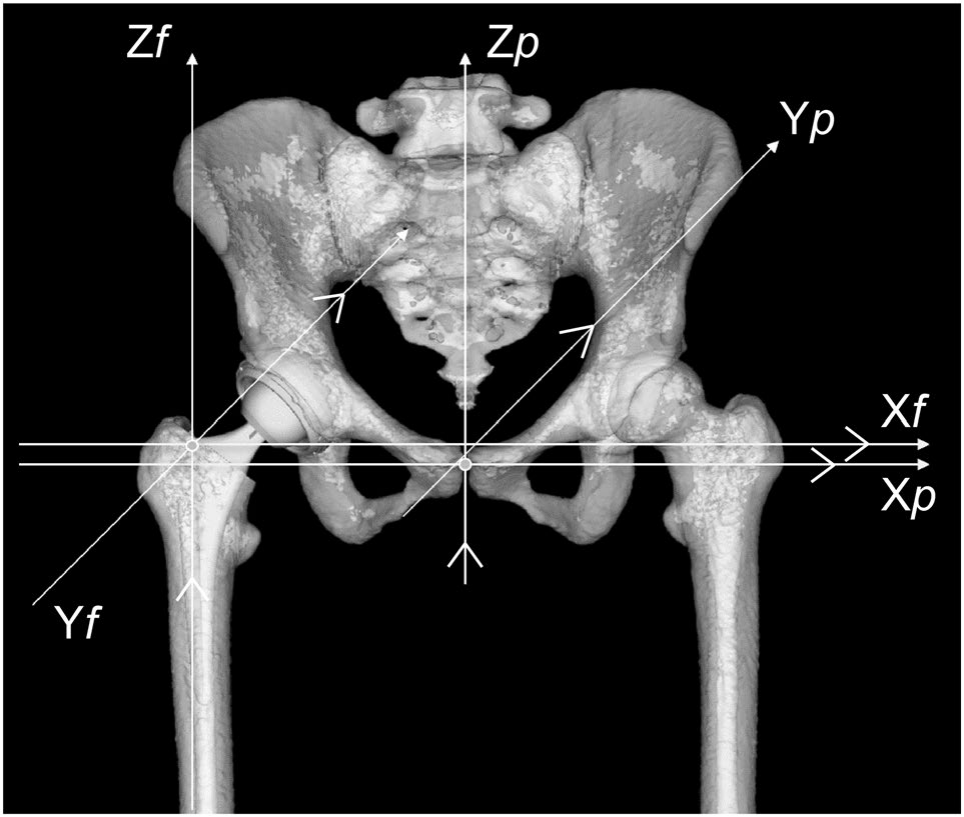
The three-dimensional model by ZedHip with the parallel coordinate system. The APP and RCP were parallel. The respective X,Y, and Z axes of the pelvis and femur coordinate system were made parallel. *APP* anterior pelvic plane, *RCP* retrocondylar plane.

**Figure 3:**
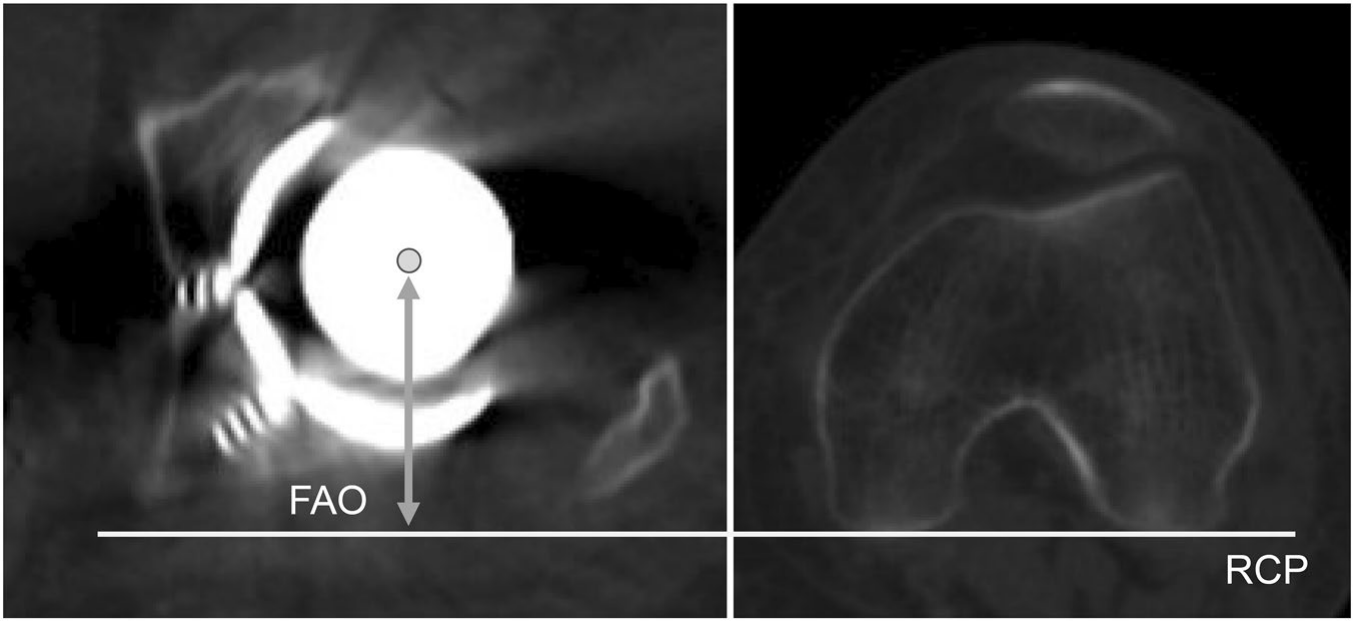
The measurement of AFO. AFO is the distance between the center of the femoral head and RCP in the axial plane. *AFO* anterior femoral offset, *RCP* retrocondylar plane.

We first investigated AO (the distance between the most distal point of the teardrop and the femoral head center) (Fig. 1), with adjustment of the 3D pelvis model to the APP using ZedHip. The 3D model of the femur was adjusted to the retrocondylar plane (RCP), as previously reported^15^. In addition, the APP and RCP were made parallel (Fig. 2). Then, FO (the distance between the femoral head center and femoral shaft axis) (Fig. 1), AFO (Fig. 3), GFO (the sum of AO and FO)^16^, and the distance in the Z-axis of the pelvis from the lower edge of the teardrop to the apex of the lesser trochanter (LLD: the difference between the surgical and non-surgical sides) (Fig. 1) were measured when the respective X,Y, and Z coordinate systems were made parallel (Fig. 2).

Especially, FO, GFO, and LLD in this study were considered as uniform values that were not affected by hip rotation. All values were corrected per 100 cm of height. Cup placement angles were evaluated by radiographic definition^9^ relative to the pelvic coordinate system (FPP), and stem anteversion was evaluated by the angle between the tangent line of the posterior condyle and the stem axis relative to the RCP.

The HHS was assessed by experienced orthopedic surgeons within 3 months preoperatively and at 1 year postoperatively. Then, the HHS was then converted to modified (m)HHS to focus more on function and activity^17^.

### Statistical analysis

SPSS statistical software, version 24 (IBM Corp., Armonk, NY USA) was used to analyze the data. The respective differences in AO, FO, AFO, and GFO on the surgical and non-surgical sides were analyzed using a paired t-test. Linear regression was used for these associations with total mHHS, pain, and function scores at 1 year postoperatively. As AO, FO, AFO, and GFO are neither too large nor too small^1^, they were also evaluated by binomial approximation, and the maximum value of any significant association was determined.

Among the significant association parameters, the cutoff value for mHHS ≥ 80, which is considered a good outcome^18^, was calculated using the receiver operating characteristic (ROC) curve with the maximum value in the binomial approximation as the center and the absolute value around it; then, the acceptable range was calculated. Regarding the paired t-test and correlation analysis, statistical power (type II (β) error) was evaluated using a post hoc analysis, with 0.5 as the effect size (d) and 0.05 as type I (α) error. We calculated intraobserver and interobserver reliabilities using intraclass correlation coefficients (ICC). One-week intervals for intraobserver reliability were measured at least twice. A *p*-value < 0.05 was considered statistically significant.

## Results

**Figure 4:**
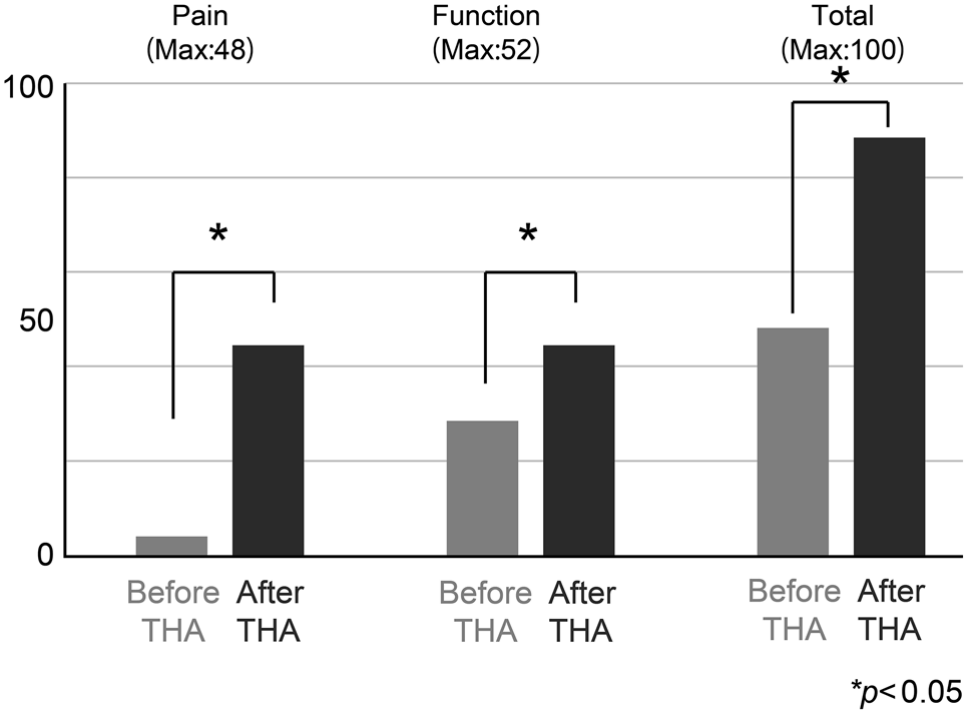
The change of the score of total mHHS, pain, and function. The total mHHS, pain score, and function score were significantly improved after THA. *mHHS* modified Harris Hip Score, *THA* total hip arthroplasty.

**Figure 5:**
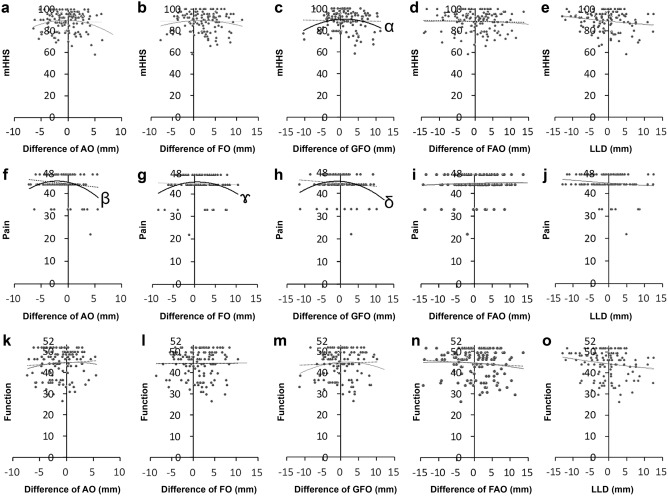
Binomial approximation and maximum value. We evaluated using binomial approximation, and the maximum value of any significant association was determined. Panels (**a**–**e**) show plots of mHHS versus the differences in AO, FO, GFO, FAO, and LLD, respectively. Panels (**f**,**g**,**h**,**i**,**k**,**l**) show plots of pain versus the differences in AO, FO, GFO, FAO, and LLD, respectively. Panels (**k**–**o**) show plots of the function versus the differences in AO, FO, GFO, FAO, and LLD, respectively. There was a significant correlation in panels (**c**,**f**,**g**,**h**). There was no significant correlation in panels (**a**,**b**,**d**,**e**,**i**–**o**).

**Figure 6:**
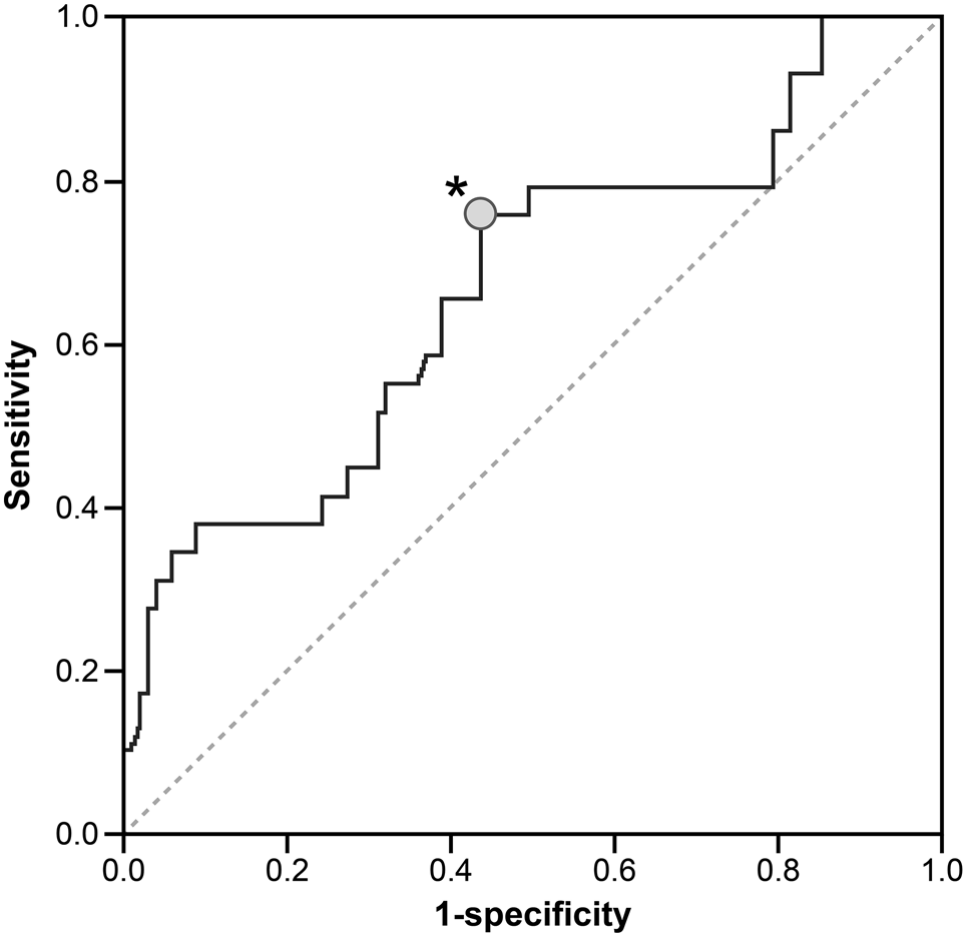
ROC and COV of GFO to be mHHS≧80. The cutoff value of mHHS ≥ 80 was calculated using the ROC. ⋆ was the cut off value. The sensitivity and 1-specificity were 0.759, and 0.437, respectively. *ROC* receiver operating characteristic curve, *COV* cut off value, *GFO* global femoral offset, *mHHS* modified Harris Hip Score.

The details of the participants are presented in Table 1. The placed angle was 40.0° ± 4.9° at radiographic inclination, 16.8° ± 6.1° at radiographic anteversion, and 26.3° ± 10.9° at stem anteversion. The mHHS scores were significantly improved from 49.0 to 88.8 points in total mHHS, from 20.0 to 44.5 points in pain, and from 28.9 to 44.4 points in function (Fig. 4). The values of AO, FO, GFO, and AFO are shown in Table 2. Significant correlations were found between the difference in AO, FO, GFO, and pain score in binominal, with maximum values of − 1.24, + 1.54, and + 0.90 mm/100 cm body height, respectively (Fig. 5f,g,h, respectively; Table 3). For GFO and mHHS in binominal, the maximum value was + 1.17 mm/100 cm body height (Fig. 5c; Table 3). Significant correlations were not observed between others (Fig. 5a,b,d,e,i–o). The cutoff value for mHHS ≥ 80 using the ROC curve was 2.92 mm; the area under the curve was 0.668, p < 0.001; sensitivity, 0.759; and 1-specificity, 0.437 (Fig. 6). The power analysis of the paired t-test and correlation showed power values of 0.941 and 0.980, respectively. Both intraobserver and interobserver reliabilities were 0.8 and more in ICC (Table 4).

**Table 2:** Measurement value of pelvic and femoral parameters.

	Surgical side	Non-surgical side	Difference between surgical and non-surgical side
AO (mm)*	19.0 ± 2.4 (11.7–25.7)^‡^	20.1 ± 1.9 (14.2–24.8)^‡^	− 1.2 ± 2.7 (− 7.3 to 5.6)
FO (mm)*	22.6 ± 3.6 (14.3–32.3)^‡^	21.2 ± 3.4 (9.5–29.1)^‡^	1.3 ± 3.7 (− 8.5 to 10.4)
GFO (mm)*	41.5 ± 4.5 (29.5–52.4)	41.4 ± 4.3 (29.6–50.1)	0.2 ± 4.3 (− 11.4 to 10.2)
AFO (mm)*	22.1 ± 4.4 (10.5–34.1)	21.6 ± 4.4 (9.0–36.2)	0.5 ± 5.5 (− 1.43 to 13.7)
LLD (mm)*			− 0.4 ± 5.1 (− 12.9 to 12.6)

Negative values indicated that the length of the surgical side was longer than that of the non-surgical side.

*AO* acetabular offset, *FO* femoral offset, *GFO* global femoral offset, *AFO* anterior femoral offset, *LLD* leg length discrepancy.

*Mean ± standard deviation (range).

These values were adjusted to 100 cm of body height.

^‡^p < 0.05.

**Table 3:** Formulae of regression equation.

	Formula	Correlation coefficient
α	y = − 0.046x^2^ + 0.095x – 45.326	*r* = 0.308
β	y = − 0.126x^2^ – 0.514x + 44.941	*r* = 0.293
γ	y = − 0.058x^2^ + 0.085x + 45.240	*r* = 0.215
δ	y = − 0.078x^2^ + 0.010x + 90.170	*r* = 0.233

**Table 4:** Reliability of the measurement values.

	Intraobserver reliability		Interobserver reliability	
	Mean absolute error	ICC	Mean absolute error	ICC
AO (mm)	1.8 ± 1.1 (0.1–3.9)^‡^	0.913, < 0.001 (0.877–0.939)*	1.9 ± 1.1 (0.1–4.2)^‡^	0.879, < 0.001 (0.828–0.914)*
FO (mm)	2.2 ± 1.2 (0.2–3.9)^‡^	0.864 < 0.001 (0.805–0.905)*	2.7 ± 1.4 (0.2–4.9)^‡^	0.840, < 0.001 (0.781–0.884)*
GFO (mm)	2.5 ± 1.6 (0.0–5.1)^‡^	0.841, < 0.001 (0.634–0.929)*	2.8 ± 1.8 (0.0–6.4)^‡^	0.835, < 0.001 (0.629–0.927)*
AFO (mm)	1.6 ± 1.1 (0.1–3.4)^‡^	0.929, < 0.001 (0.899–0.950)*	1.8 ± 1.2 (0.1–4.0)^‡^	0.891, < 0.001 (0.846–0.923)*
LLD (mm)	1.9 ± 1.5 (0.0–5.6)^‡^	0.889, < 0.001 (0.859–0.920)*	2.6 ± 1.6 (0.0–6.7)^‡^	0.867, < 0.001 (0.817–0.904)*

*ICC* intraclass correlation coefficient, *AO* acetabular offset, *FO* femoral offset, *GFO* global femoral offset, *AFO* anterior femoral offset, *LLD* leg length discrepancy.

^‡^Mean ± standard deviation (range).

*ICC, *p*-value (95% confidential distance).

## Discussion

Our results showed that the outcome following THA was favorable when the GFO was 1.17 ± 2.92 mm/100 cm of BH, that is, the optimal range of the difference of GFO was from − 1.75 to 4.09 mm/100 cm of BH. Especially, the optimal difference of GFO was from − 2.8 to 6.54 mm when the BH of the patient was 160 cm, similar to the report of Mahmood et al., in which the optimal difference of GFO was within 5 mm^1^. We speculated that when FO with or without GFO was too short compared to the non-surgical side, the abductor muscle strength, walking distance, and speed decreased; consequently, patients may experience pain related to fatigue of the muscle around the hip joint^2,19^. In contrast, in the case of FO with or without GFO made too long, pain at the lateral aspect of the hip joint, known as “greater trochanteric pain syndrome”, may occur^20^.

Conversely, the pain score was better when the AO was smaller than that of the non-operative side. When the AO was larger on the surgical side, that is, when the cup was placed laterally, the cup protruded from the acetabular rim, causing friction between the iliopsoas and the rim of the cup, possibly leading to pain. In addition, FO, FAO, and LLD were not associated with total mHHS, pain, or functional scores. This may be attributed to the fact that the difference between the surgical and non-surgical sides was not large in this study.

Our study is notable in that both the pelvis and the femur were assessed by alignment to a certain coordinate system; to our knowledge, this was the first report using this 3D method. When the studied parameters are assessed using plain radiographs, FO may not be accurate because of the differences in hip rotation^5^. In addition, adduction and abduction of the hip can affect LLD. Therefore, it is difficult to directly reflect the results obtained from the radiographic assessment for preoperative planning, including stem anteversion, which can affect the offset. Therefore, this study is the first report, in which these factors were considered and assessed, and our results seem to have a high clinical value.

This study has some limitations. First, only 140 participants were enrolled. Second, this was a retrospective, cross-sectional study. Furthermore, assessment was made using the mHHS. Our results revealed that functional scores were related to all the studied parameters. It is possible that there were differences in function that could not be represented by the mHHS; therefore, additional research is necessary. It may be possible to further limit the range of optimal GFO by using a clinical score instead of the mHHS for evaluation. Even considering these limitations, to our knowledge, this study is the first report to measure the parameters by this 3D method. Furthermore, the obtained results imply that the use of this method may improve the outcome after THA. In future studies, we would like to examine the optimal offset for bilateral hip OA.

## Conclusion

For THA, the clinical outcome was the best when the AO and FO differences from the non-surgical side were − 1.24 mm, + 1.54 mm/100 cm of BH, and GFO 1.17 ± 2.92 mm/100 cm of BH, respectively. We measured these parameters by aligning both the pelvis and the femur to a certain coordinate system for assessment. To our knowledge, this is the first report using this 3D method. The results could lead to improved preoperative planning for THA and subsequently improved postoperative outcomes.

## Data Availability

The datasets generated during and/or analyzed during the current study are available from the corresponding author on reasonable request.
